# Ohio State Dental Society

**Published:** 1881-01

**Authors:** 


### *Ohio State Dental Society.*

The following was adopted, by a rising vote, at Columbus, December 9, 1880, and ordered sent to all the dental journals.

W. H. SILLITO,
*Rec. Secretary.*

### IN MEMORIAM.

Since our last annual meeting, and shortly after its adjournment, the practitioners of dentistry throughout the civilized world were shocked and saddened by the announcement of the death, at Paris, France, from congestion of the brain, of our esteemed friend and co-laborer, DR. SAMUEL S. WHITE.

Although distant from home, his last hours were cheered and the bitterness of death was mitigated through the loving ministrations of a son and daughter—who had accompanied him.

When the cable transmitted the intelligence of his death, many hearts were burdened with grief in sympathy with his bereaved family.

No words eulogizing Dr. White are needed here. His career is known to us all—a noble life. The sterling character of the man ; the uniform worthiness of his aims ; his energy and industry ; his conscientious, earnest and persistent endeavors to benefit the dental profession, his upright and generous dealing, furnished an example worthy of imitation by us all. A record of his manliness and worth is engraved upon the hearts of all who knew him, and deepest in the memories of those who knew him best.

Cherishing appreciative and affectionate remembrances of our personal and professional relations with Dr. White, we desire to assure his family of our unfeigned sympathy with them in their affliction, and place on record this testimonial of a sincere esteem and love for our departed friend.

F. H. Rehinkle,
J. Taft,
G. W. Keely,
*Committee.*

---

